# The reliability of malaria rapid diagnostic test kit for detecting *P. falciparum* PfHRP2 in dried blood spot samples preserved under different conditions and durations

**DOI:** 10.1186/s13104-025-07613-5

**Published:** 2025-12-12

**Authors:** Enoch Aninagyei, Joshua Apekey, Comfort Addo Boatey, Emmanuel Banono, Jude Obuobi Adu, Adjoa Agyemang Boakye

**Affiliations:** Department of Biomedical Sciences, School of Basic and Biomedical Sciences, University of Health and Allied Sciences, PMB 31 Ho, VR Ghana

**Keywords:** Malaria, Dried blood spot, Storage, Room temperature, *Plasmodium falciparum* histidine-rich protein 2

## Abstract

**Objective:**

This study investigated the reliability of the malaria rapid diagnostic test (mRDT) kit for detecting *Plasmodium falciparum* histidine-rich protein 2 (PfHRP2) in dried blood spot samples preserved under different conditions and durations.

**Results:**

Sixty whole blood samples (approx. 750 mL), which contained the PfHRP2 protein, were collected from patients with uncomplicated malaria. Fifteen dried blood samples (DBS) were made from each sample, making a total of 900 DBS. A set of five DBS each were stored at room temperature (25 °C), 6 °C and – 60 °C, for 30, 60, 90, 120 and 150 days. PfHRP2 was detected in each stored DBS sample. Irrespective of sample storage conditions, PfHRP2 was reproducible from day 30 to 120. However, after storage for 150 days, the positivity rate reduced to 90%, 88.3% and 80% (*p* = 0.236) when samples were stored at room temperature, 6 °C and – 60 °C, respectively. Although the positivity rates were statistically similar, room temperature offers a cost-effective, equipment-free, and reliable method for preserving PfHRP2 for up to 120 days.

**Supplementary Information:**

The online version contains supplementary material available at 10.1186/s13104-025-07613-5.

## Introduction

*Plasmodium* parasites cause malaria, which is a life-threatening disease. *Plasmodium falciparum* is known to be the most common species found worldwide and causes the most lethal form of malaria. As a result, an early diagnosis could save a life [29]. Malaria is far more prevalent in Sub-Saharan Africa than elsewhere in the world. Microscopy has traditionally been the primary approach for diagnosing malaria in health facilities, although polymerase chain reaction (PCR) gives appropriate sensitivity in well-equipped research and reference institutions. A systematic review by Yalley et al. [31] and Moody [21] found that malaria microscopy has limited sensitivity, and its effectiveness is also dependent on the operator. On the other hand, PCR was found to be not amenable to resource-limited areas, where malaria is endemic [25]. These demerits of microscopy and PCR led to the invention of malaria rapid diagnostic test kits [27]. As of the year 2018, 35 mRDT kits manufactured by 17 companies have been assessed and approved by the World Health Organization [27].

Malaria rapid diagnostic tests (mRDTs) are gaining popularity for the diagnosis of malaria due to their increased sensitivity (Opoku et al., [24]), user-friendliness, rapid turnaround time, low operational costs and amenability to different ing settings. MRDT could also aid in parasite speciation when specific proteins are detected [17]. The common *Plasmodium falciparum* antigen detected by most mRDT kits is the *P. falciparum* histidine-rich protein 2 (PfHRP2) [27]. In Ghana, *Plasmodium falciparum* causes over 95% of malaria cases [4]. The PfHRP2 protein is specific to *P. falciparum* [6]. The protein is deposited at the surface of the invaded red blood cell [22], during the erythrocytic stage of the *P. falciparum* life cycle. Therefore, the detection of the PfHRP2 protein in blood is akin to malaria diagnosis. Although malaria microscopy and molecular techniques such as PCR are the gold standards for detecting *Plasmodium* spp., these methods require electricity and trained personnel, making mRDT kits the preferred choice. Additionally, microscopy has limited sensitivity [24], and its effectiveness is dependent [11]. Conversely, PCR is an expensive and sophisticated technique [12] with a long turnaround time [15].

Malaria RDT kit manufacturers prescribe whole blood as the sample of choice for optimum kit ing accuracy. However, due to the desire for bulk processing of clinical and field samples and the unavailability of mRDT kits, sample storage is inevitable. It has been reported that storing whole blood in an anticoagulant between 4 and 6 °C for a long time, especially in EDTA anticoagulant, results in sample haemolysis and subsequent degradation [30]. In addition, Kift et al. [18] have reported that − 80 °C storage facilities adequate to store samples for a long time, however, this facility is not available in resource-constrained laboratories, due to its cost price and operation cost. Therefore, it is important to explore short to medium-term storage of dried blood spot (DBS) samples containing the parasite proteins (PfHRP2) to determine the length of storage and under which storage conditions the detection of the parasite proteins will be reproducible. DBS has been used to store malaria samples for several molecular studies [1, 7, 14]. However, the stability of PfHRP2 in stored DBS has not yet been evaluated.

## Materials and methods

### Study design and locations

This laboratory-based experimental study was carried out on DBS samples containing *P. falciparum* parasites. The DBS was prepared with Whatman Grade 31 ET CHR (China) filter paper. Whole blood samples were collected from patients selected from three health facilities from southern Ghana, namely, Ngleshie-Amanfrom Polyclinic (*n* = 15), Bososu Health Center (*n* = 20) and Catholic Hospital, Anfoega (*n* = 25). The laboratory analysis was done in the Parasitology and Immunology Laboratory in the Department of Biomedical Sciences, University of Health and Allied Sciences, Ho, Ghana.

### Sample collection

In all, 4 mL of venous blood samples were collected from 60 patients with uncomplicated malaria (initially screened with mRDT and subsequently confirmed with microscopy). Blood samples were collected from the patients with malaria within one week. The microscopy and the rapid testing were performed by the National Malaria Elimination Control, Ghana Certified Medical Laboratory Scientists, available in the study health facilities. Only blood samples with strong positive test bands were selected for this study.

### Laboratory procedures

On the day of collection (day 0), the samples were spotted on filter paper. About 750 µL of blood was used to prepare 15 DBS for each sample and air dried, at room temperature (25 °C) for about 5 h. In all, 900 DBS were stored and tested. Five of the DBS were stored in a separate Ziploc bag at room temperature (25 ℃), 6 ℃ and – 60℃ for up to 150 days. On each day of sample analysis, sample hemolysate was prepared by adding 100 µL of phosphate-buffered saline (pH = 7.2) to two 6 mm DBS punch in a 2 mL Eppendorf tube. The setup was incubated at room temperature for 15 min, followed by a brief vortexing.

### Detection of PfHRP2 in the haemolysate

*P. falciparum* HRP2 was detected by using the First Response malaria rapid diagnostic test kit (Premier Medical Cooperation Private Ltd, India). Before testing, the mRDT kits were stored at room temperature (approx. 25 °C), as recommended by the manufacturer. The First Response detects the PfHRP2 proteins, which are exclusive to the *P. falciparum*. According to the last WHO assessment round 8 of mRDT kits [27], the kit was found to have a sensitivity of 95% for parasitemia of 200 parasites/µL and 100% when parasitemia of 2000 parasites/µL was tested. Briefly, 5 µL of the sample hemolysate was dispensed at the sample window of the test, followed by two drops of the sample buffer at the appropriate well. Results were read 20 min after the start of the test. Tests with two red lines at both the control and the test band were considered positive, whereas tests with only one red band at the control band were considered negative. Only one line at the test band without one at the control band was a false positive test. Test outcomes were classified as strong positive (when the test band is visibly pinkish), weak positive (when the test band is light pinkish) and very weak positive (when the test band can barely be seen).

### Data analysis

The PfHRP2 positivity rates for storage days 30, 60, 90, 120 and 150 were obtained by dividing the time point positivity rates by the total number of samples analysed (*n* = 60). In addition, a graphical plot of positivity rate at different temperatures, with time of follow-up was generated. Further, the positivity rates were compared using Chi-square; p-values less than 0.05 were considered statistically significant, at 95% confidence interval. Finally, a heatmap for the RDT positivity for all 60 samples at different temperatures and times was developed.

## Results

### Baseline characteristics of the samples analysed

The parasite densities of the 60 blood samples ranged from 968 to 65,871 parasites/µL of blood. The samples were obtained from 39 females (65%) and 21 males (35%). Children accounted for 40% (24/60) and adolescents for 28.3% (17/60), making both of them the majority participants. Most of the participants presented with chills (44, 73.3%), fever (55, 91.7%), and headache (39, 65%). Others experienced vomiting (21, 35%), convulsions (17, 28.3%), and pallor (21, 35%). The mean haemoglobin level was 9.8 g/dL (± 1.7 standard deviation) (Supplementary file 1). In total, 900 DBS samples prepared from the 60 samples were stored and followed up.

**Fig. 1:**
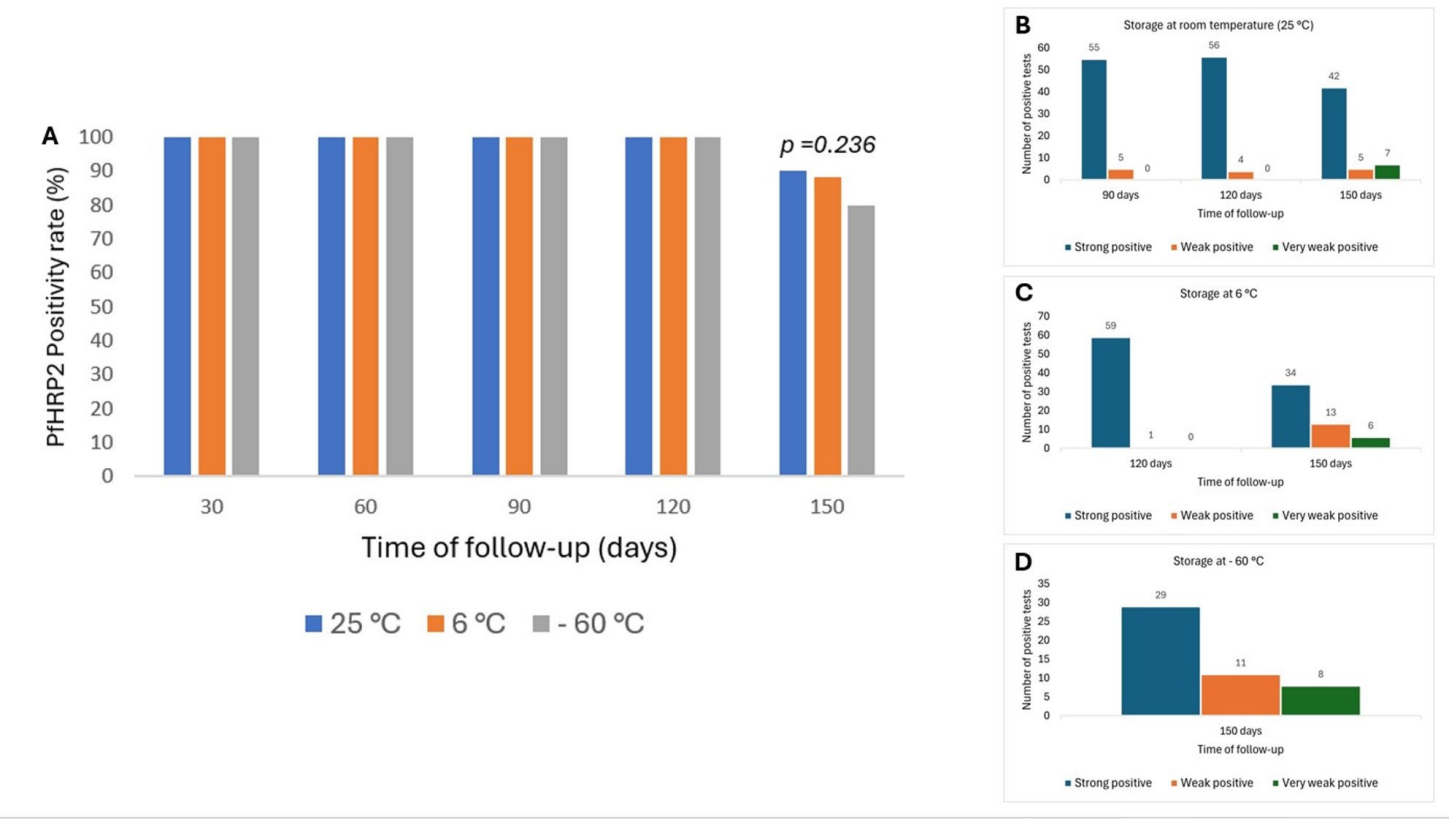
Graphical representation of follow-up positivity rates

All the baseline samples were strongly positive (Fig. 1).

### Analysis of PfHRP2 positivity results by storage duration and temperature

**Fig. 2:**
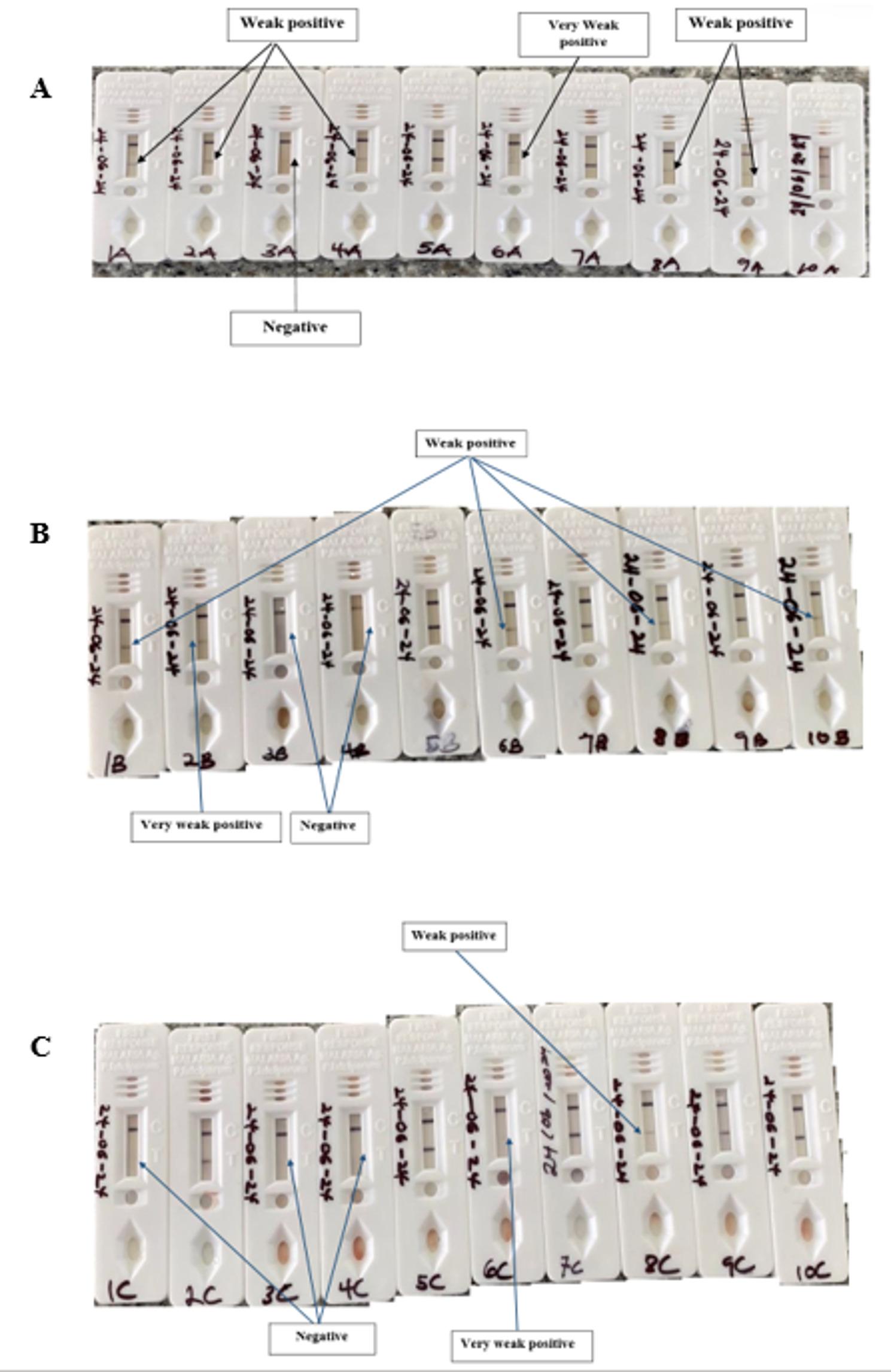
Malaria RDT results for samples 1–10 stored at day 150. **A** DBS stored at room temperature, (**B**) DBS stored at 6 °C, (**C**) DBS stored at − 60 °C. Parasite counts (/µL) for the samples were sample 1 (15981), sample 2 (1671), sample 3 (1013), sample 4 (17114), sample 5( 35817), sample 6 (29711), sample 7 (45012), sample 8 (65871), sample 9 (33667), sample 10 (15001)

When the DBS samples with PfHRP2 were stored for 30–120 days, the positivity rates were the same (100%), irrespective of the storage temperature. However, the positivity rate dropped to 90%, 88.3% and 80% when the DBS samples were stored at room temperature, 6 °C and – 60 °C, respectively. However, the differences in the positive rates were not statistically significant (*p* = 0.236) (Fig. 1A). Even though the positivity rates were the same for storage days 30–120, there were differences in the intensity of the test bands. At room temperature, five of the 60 samples changed from strong positive to weak positive at day 90, whilst at day 120, four of the tests, which were previously strongly positive, were weakly positive (Fig. 1B). At 6 °C and – 60 °C storage, one sample was weakly positive at day 120 (Fig. 1C). At storage day 150, 5 (room temperature), 13 (6 °C) and 11 (− 60 °C) samples turned weakly positive whilst 7, 6 and 8 samples turned very weakly positive when stored at room temperature, 6 °C and − 60 °C, respectively (Fig. 1B–D). The mRDT results, as shown on the test cassette, for the first 10 samples are shown in Fig. 2.

### Individual sample reactions at different temperatures and times

**Fig. 3:**
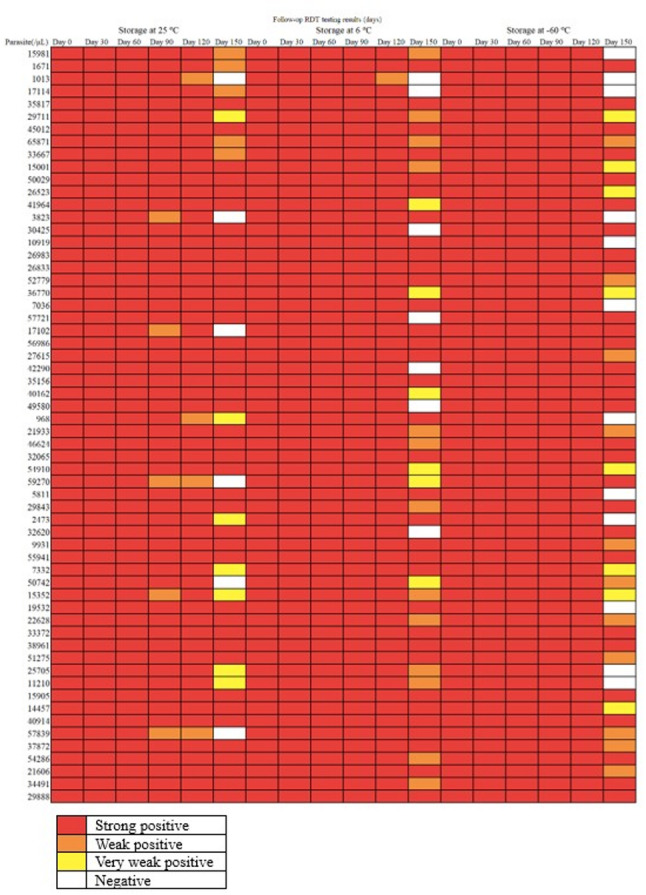
Heatmap of the positivity rates of the baseline and the follow-up testing. The red, amber, yellow, and the while cell colours represent strong, weak, very weak positive and negative, respectively

Figure 3 illustrates the heatmap depicting the appearance of individual tests during the follow-up assessment. The samples exhibited variable bands when stored at different temperatures and durations. The first sample, with a parasite count of 15,981 /µL, demonstrated a strong reaction akin to the baseline reaction observed from days 30 to 120, regardless of the storage conditions. However, the band weakened at day 150 when stored at room temperature and 6 °C, and it was negative when stored at -60 °C for 150 days. Another sample, with a parasite count of 1671 /µL, displayed strong bands at both 6 °C and − 60 °C during storage but was weakly positive at day 150 when kept at room temperature. Some samples, including those with counts of 10,919, 56,986, 35,156, and 50,029 /µL, remained strongly positive throughout, irrespective of the storage conditions. Other samples, such as the one with 59,270 parasites /µL, turned weakly positive at days 90 and 120 but negative at day 150 when stored at room temperature, while remaining strongly positive throughout storage at 6 °C and − 60 °C, except for being very weakly positive at day 150 under 6 °C storage.

### Test bands intensity of samples with low baseline parasite counts reduced during storage

Generally, it was found that the samples that changed from strong positive to negative at a certain time during storage had low parasite counts (mean = 8818 parasites/µL) compared to samples that turned weak (mean = 17111 parasites/µL) and very weak positive (mean = 12352 parasites/µL). The samples that were strongly positive at baseline and during the follow-up had a higher mean baseline parasite count (22808 parasites/µL).

## Discussion

Sample storage is necessary in laboratory practice for bulk processing, unavailable reagents, or technology issues requiring samples to be stored or shipped abroad for analysis. This study found that PfHRP2 was detectable in all the DBS samples stored at room temperature, 6℃ and – 60℃ for up to 120 days. The observation at room temperature could be explained by the dried blood spot samples containing little to no water molecules, which in turn limited protein degradation. Aqueous solutions have been reported to play an invaluable role in governing the structure, stability, dynamics, and function of proteins [19]. However, interparticle interactions dictate the thermodynamic stability of aqueous protein solutions, and this can in some instances lead to changes in protein conformation, which result in the loss of protein function [5]. Thus, in most cases, protein degradation occurs at room temperature when samples are stored in an aqueous medium [3, 8]. However, when blood spots are adequately dried on filter paper, protein components are stable at room temperature for at least one year [10].

During storage, some reaction bands shifted from clear to weak or very weak, suggesting partial protein loss, whereas the remaining proteins preserved their structural integrity. Previous study reported that proteins could be stabilized at temperatures between 2 and 6 °C [26] to sub-zero temperatures for a short time [9]. These temperatures are enough to halt or prevent proteolytic degradation of the proteins. This explains why PfHRP2 was preserved and remained detectable when the samples were stored at 6 °C and − 60 °C for up to 120 days. Thus, for every protein, it is important to define its optimum temperature to identify the best storage conditions for it.

In addition, the study found that protein loss increased at − 60 °C when the samples were stored for 150 days. This could be due to higher protein loss or degradation (20%) at − 60 °C compared to when the samples were stored at 6 °C (11.7%) and room temperature (10%), even though the differences in the protein loss were not significant. The loss of some parasite proteins at room temperature was due to the marginal stability of proteins at higher temperatures [2]. During prolonged storage at 6 °C, proteins can adopt non-native conformations [16] and may lose their functions. Prolonged sample storage at – 60 °C is likely to negatively affect protein integrity in several ways, such as lipid oxidation, protein denaturation, protein browning and physical damage from ice crystal formation [23]. Ice crystals in the interstitial spaces may perturb the protein tertiary fold [20] which may lead to loss of protein structure and subsequently function. These processes could change the protein conformation, integrity and function, and that may explain in part why the positivity rate decreased by up to 20% during prolonged storage at − 60 °C. Additionally, low temperatures can lead to the hydration of polar and non-polar groups of proteins, thereby decreasing the hydrophobic interactions [13]. Because hydrophobic interactions play a crucial role in protein folding, these disruptions can induce changes in protein structure, thereby affecting their detection since the detection methods are based on recognition of the 3-dimensional structure of the protein.

The changes in the test band intensities and reduction in the positivity rates were likely to be influenced by the baseline parasite counts. The higher the baseline parasite counts, the more likely it is that the sample will remain positive during storage. A WHO report in 2019 [28] confirmed that mRDT sensitivity is constantly dependent on the concentration of target PfHRP2 present in the sample and so varies with parasite density. Sensitivity, therefore, increases with parasite density but decreases when densities fall below 200 parasites/µl.

## Conclusion

The PfHRP2 in DBS can be stored at room temperature, 6℃, and − 60℃ for up to 120 days. However, the positivity rate decreased at day 150 to 90% at room temperature storage and 88.3% and 80%, respectively, at 6℃ and − 60℃. For PfHRP2 testing within 120 or 150 days, room temperature storage is preferred due to its cost-effectiveness and protein integrity preservation.

### Limitation

The findings reported in this study are limited to the sensitivity of the First response malaria RDT kit used to detect the PfHRP2 and the assumption that the temperature of each storage condition did not significantly fluctuate. In addition, PfHRP3 could confound these findings due to its cross-reactivity with HRP2 antibodies in the RDT.

## Supplementary Information

Below is the link to the electronic supplementary material.

Supplementary Material 1.

## Data Availability

All study data collected in this study are presented in this publication.
