# Supplementary material for: The reliability of malaria rapid diagnostic test kit for detecting P. falciparum PfHRP2 in dried blood spot samples preserved under different conditions and durations
Source: BMC Res Notes. 2025 Dec 12;19:23. doi: 10.1186/s13104-025-07613-5 (PMC12817724; doi:10.1186/s13104-025-07613-5)
Supplement: Supplementary file 1 — Supplementary Material 1. [file 13104_2025_7613_MOESM1_ESM.docx]

Supplementary file 1: Demographic and clinical details of the study participants

| Variables | Total (%) |
| --- | --- |
| Demographic |  |
| Developmental stage ^a^ | |
| Child | 24 (40) |
| Adolescent | 17 (28.3) |
| Adult | 19 (31.7) |
| Gender |  |
| Female | 39 (65) |
| Male | 21 (35) |
| Tribe |  |
| Akan | 13 (21.7) |
| Ewe | 11 (18.3) |
| Krobo/Ga Adamgbe | 17 (28.3) |
| Northern descent | 19 (31.7) |
| Marital status |  |
| Married | 9 (15) |
| Unmarried adults | 14 (23.3) |
| Below marital age ^b^ | 37 (61.7) |
| Formal education |  |
| Pre-school ^c^ | 18 (30) |
| In school | 35 (58.3) |
| Completed | 7 (11.7) |
| Religion |  |
| Christian | 49 (81.7) |
| Islam | 9 (15) |
| Traditional | 2 (3.3) |
| Occupation |  |
| Government worker | 3 (5) |
| Self-employed | 8 (13.3) |
| Unemployed ^d^ | 49 (81.7) |
| Clinical |  |
| Presentations |  |
| Chills | 44 (73.3) |
| Fever | 55 (91.7) |
| Headache | 39 (65) |
| Vomiting | 21 (35) |
| Convulsion | 17 (28.3) |
| Pallor | 21 (35) |
| Haemoglobin levels |  |
| Mean level | 9.8 g/dL |
| Standard deviation | 1.7 |
| Parasitaemia (/µL) |  |
| Min-Max | 1013 - 65871 |
| Mean | 30621 |

*^a^ Child (1 to 12 years), Adolescent (**13 to 17 years), Adult (**18 to 65 years), Older adult (> 65 years); ^b^ Below marital age, if* *< 18 years; ^c^ Below formal education age (< 4 years); ^d^  including children below employable age*
